# Fabrication of a Ti porous microneedle array by metal injection molding for transdermal drug delivery

**DOI:** 10.1371/journal.pone.0172043

**Published:** 2017-02-10

**Authors:** Jiyu Li, Bin Liu, Yingying Zhou, Zhipeng Chen, Lelun Jiang, Wei Yuan, Liang Liang

**Affiliations:** 1 Guangdong Provincial Key Laboratory of Sensor Technology and Biomedical Instrument, School of Engineering, Sun Yat-Sen University, Guangzhou, PR China; 2 School of Mechanical and Automotive Engineering, South China University of Technology, Guangzhou, PR China; Helsingin Yliopisto, FINLAND

## Abstract

Microneedle arrays (MA) have been extensively investigated in recent decades for transdermal drug delivery due to their pain-free delivery, minimal skin trauma, and reduced risk of infection. However, porous MA received relatively less attention due to their complex fabrication process and ease of fracturing. Here, we present a titanium porous microneedle array (TPMA) fabricated by modified metal injection molding (MIM) technology. The sintering process is simple and suitable for mass production. TPMA was sintered at a sintering temperature of 1250°C for 2 h. The porosity of TPMA was approximately 30.1% and its average pore diameter was about 1.3 μm. The elements distributed on the surface of TPMA were only Ti and O, which may guarantee the biocompatibility of TPMA. TPMA could easily penetrate the skin of a human forearm without fracture. TPMA could diffuse dry Rhodamine B stored in micropores into rabbit skin. The cumulative permeated flux of calcein across TPMA with punctured skin was 27 times greater than that across intact skin. Thus, TPMA can continually and efficiently deliver a liquid drug through open micropores in skin.

## Introduction

Transdermal drug delivery has many advantages for the patient due to its non-invasive nature, easy accessibility, immune-surveillance functions, avoidance of first pass metabolism and prevention of gastrointestinal degradation [1–4]. However, skin, especially the stratum corneum layer, has evolved to impede the flux of toxins into the human body and minimize water loss, which indicates that it naturally has a low permeability to deliver the foreign macromolecular drugs [4–6]. Macromolecular drug delivery across the skin has primarily employed hypodermic needles, which have disadvantages, including accidental needle-sticks, risk of infection, bleeding, pain and needle phobia [7–10]. Several alternative methods have been developed to overcome the stratum corneum barrier and enhance the permeation of drug transdermal delivery, including iontophoresis [11, 12], sonophoresis [13–16], magnetophoresis [17–19], electroporation [20–22], and laser [23–26]. These methods suffer from considerable limitations in practical and economic terms [1]. Microneedle arrays (MA) are minimally invasive tools that bypass the stratum corneum barrier, accessing the skin microcirculation and achieving drug delivery by the transdermal route [1]. The use of MAs is one of the most promising delivery systems in the last decades due to its unique advantages, including pain-free delivery, minimal skin trauma, lack of bleeding or introduction of pathogens, and ease of disposal [1, 27].

Microneedle arrays applied in transdermal drug delivery can mainly be divided into the following three types: solid, hollow and porous MAs [2]. Solid MAs can deliver the drug in three approaches, including “poke and patch”, “coat and poke” and “poke and release” [1, 2, 28, 29]. Solid MAs were fabricated by various methods, such as lithography and etching [30], photolithography [31, 32], micro molding [33, 34], drawing lithography [35, 36], micromachining [34], laser cutting and so on. Solid MAs can be easily mass fabricated at a relatively low cost, but they have no continuity because they can support the delivery of a limited dose of drug, even in the case of “poke and patch” due to the fast closure of the poke pores in skin. [37, 38]. Hollow MAs can continuously transfer drugs into the skin via its holes, providing an unlimited dose of drugs. Its drug delivery approach is “poke and flow” [2]. They are mostly fabricated by the photolithographic method on silicon [39, 40]. Each hollow microneedle with one channel has a complex and high cost fabrication process. Porous MAs have many randomly distributed pores that can contain either a liquid or dry drug formulation. The liquid drug can be loaded into the pores and diffused from the microneedle matrix into the skin. The interconnecting holes guarantee continuous drug delivery. The dry drug can be stored in the pores, hydrated with the interstitial fluid, and diffused into skin. Thus, porous MA can facilitate transdermal drug delivery in three approaches, including “poke and patch”, “poke and release”, and “poke and flow”. Therefore, porous MA is a flexible and multifunctional transdermal drug delivery system.

However, research on the fabrication and drug delivery performance of porous MA receives less attention due to its complex fabrication process and easy fracture in comparison with solid and hollow microneedles. Silicon, polymer, ceramic and metal can be employed to fabricate porous MA. M. Shirkhanzadeh [41] electrochemically coated porous calcium phosphate on the tips of stainless steel microneedles to deliver the solid trehalose. Ji et al. [42, 43] and Chen et al. [44] fabricated the porous tips of silicon MA by electrochemical etching for high molecular weight drug delivery. The above porous MA consisted of partly porous structures and cannot continuously deliver liquid drug. Park et al. [45] ultrasonically welded Poly (lactic acid) micro-particles to fabricate porous MA in the applications of biosensing and tissue engineering. However, the polymer porous MA was fragile and unable to penetrate the skin. Liu et al. [39] fabricated a porous polymer MA by photopolymerization of an acrylate monomer in the presence of a porogen within a mold for the rapid fluid transport. It is a simple fabrication method and porous MA has sufficient strength to penetrate the skin, but this research has focused little on the drug delivery. Alumina ceramic porous MA was mostly produced using a micromolding technique by ceramic slurry cast into micromold for drug and vaccine delivery [2, 46–48]. Micromolding techniques offer the advantage of being able to develop device production as a low cost process due to the potential for up-scaling the technology [10]. The alumina porous MA shows good chemical and compression resistance, but it is brittle and easily fractured during manual skin insertion [47]. Porous metal has good mechanical strength, which was employed to fabricate Ti porous MA. Yan et al. [37] fabricated Ti porous MA by an integrated process of wire-electrode cutting and wet etching from a porous Ti wafer for the injection of insulin. This fabrication process is costly and not suitable for mass production of porous MA.

Here, a modified metal injection molding (MIM) method was proposed to fabricate titanium porous microneedle array (TPMA) for transdermal drug delivery. Titanium has good biocompatibility and excellent mechanical strength for porous MA [37, 49]. The MIM fabrication method is simple and suitable for mass production. In the subsequent work, the fabrication process of TPMA will be discussed, morphology of TPMA will be characterized by SEM, penetration performance will be tested by a self-developed setup, and dry drug diffusion and liquid drug permeation will be investigated in vitro.

## Materials and methods

### Ethics statement

This study was performed in strict accordance with the recommendations in the Guide for the Care and Use of Laboratory Animals of the National Institutes of Health. The protocol was approved by the Institutional Animal Care and Use Committee (IACUC), Sun Yat-sen University (Approval Number: IACUC- DD-16-0901). All efforts were made to minimize suffering. The penetration experiments were tested on three healthy volunteers aged from 23 to 26 at the room temperature. The study was approved by the ethics committee of the Work Injury Rehabilitation Center of Guangdong Province (approval number: AF/SC-07/2016.29). All volunteers provided written informed consent.

### Experimental preparations

Pure titanium powder (average diameter: 5 μm, Shanghai ST-Nano science and technology co., LTD China), poly (vinyl butyral) (B-98, Aladdin), benzyl butyl phthalate (B109815, Aladdin), ethanol (98 wt. %, Aladdin), solsperse 20000 (Ur-Techchemicals, Inc. China), and Rhodamine B (R104961, Aladdin) were purchased. A titanium slurry was prepared for the casting. Titanium (46 wt. %) was suspended in 46 wt. % ethanol containing 1 wt. % poly (vinyl butyral) as a binder, 6.4 wt. % butyl benzyl phthalate as a plasticizer, and 0.6 wt. % Solsperse 20000 as a dispersant.

Fresh rabbit skin was always employed for the transdermal drug delivery experiment in vivo [50–52]. A New Zealand rabbit (male, 3 months old, 3.0 Kg) was purchased from XinHua experimental animal farms (Huadu District, Guangzhou City, China). All procedures followed the guidelines of the Institutional Animal Care and Use Committee (IACUC), Sun Yat-sen University. The rabbit was mercy killed by pentobarbital through intravenous injection. The hair on the skin was removed. The skin was cut into squares in the size of 20 mm × 20 mm with an average thickness of 2.5 ± 0.1 mm.

### Fabrication process

**Fig 1:**
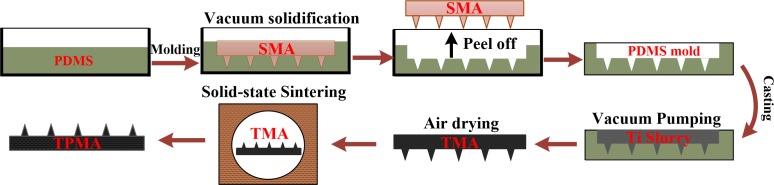
Fabrication process sketch for TPMA.

Fig 1 shows the fabrication process steps of TPMA. TPMA was fabricated by the modified MIM. (1) PDMS mold of MA was fabricated by the replication method. A stainless steel microneedle array (SMA) (Lanyou Biological technology co., LTD, China) was purchased as a pattern. SMA is cuboid with its size of 10 mm×10 mm×2 mm, 6×6 microneedle array, 500 μm microneedle height, and 1 mm inter distance. The PDMS mold was replicated, vacuum solidified and released using SMA.

**Fig 2:**
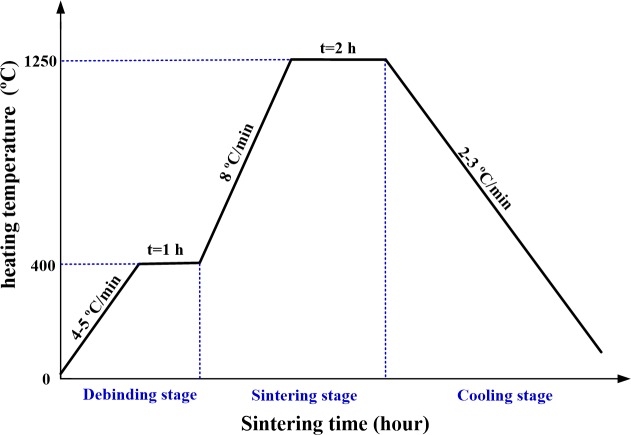
Solid-state sintering process curve for TPMA.

(2) The titanium microneedle array (TMA) green body was fabricated by a micro-molding method. The slurry prepared was poured into PDMS mold and vibrated by ultrasound, and air bubbles were removed by vacuum pumping in a vacuum chamber (PC-3, Yueci Electronic technology co., LTD, China) for 15 min. TMA green bodies were air-dried at room temperature for approximately 48 h and released from the PDMS molds when the alcohol was completely evaporated.

(3) TPMA was fabricated by the solid-state sintering method. TPMA was sintered in a tube sintering furnace (FWL (ZK) 08-70-3, FaceROM, China) under protection atmosphere. Argon gas was continuously blown during the sintering process. The solid-state sintering process curve for TPMA is presented in Fig 2. The sintering program included 1 h at 400°C with a heating up rate of 4–5°C/ min under Ar atmosphere of 20 ml/min, 2 h at 1250°C with a heating up rate of 8°C/min, and furnace cooling to 100°C with a cooling rate of 2–3°C / min.

### Characterization

The morphology of TPMA was observed by SEM (Quanta 400F, OXFORD, Holland). The height of microneedle was measured by an optical microscope (VHX-2000, Keyence, Japan). The pore size distribution of TPMA was measured by the mercury intrusion method (PoreMaster-60, Quantachrome, America). The element distribution of TPMA was investigated by EDS (Quanta 400F, OXFORD, Holland).

### Penetration performance

**Fig 3:**
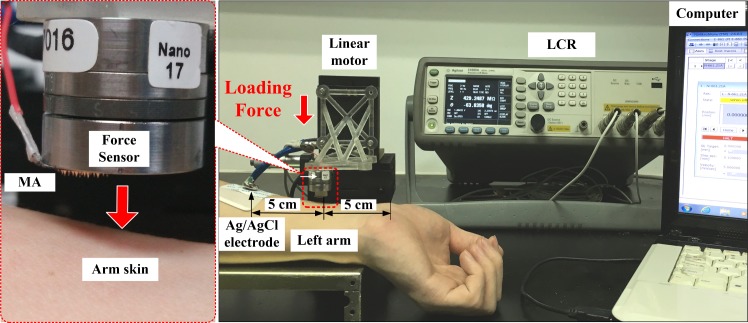
Setup for penetration performance test of TPMA.

The microneedle array should penetrate the extraordinary barrier layers of skin, especially the stratum corneum, to deliver macromolecular drugs [30]. As a result, the penetration performance of TPMA needs to be investigated. A setup was designed to test the penetration performance of TPMA, as shown in Fig 3. The left inner forearm skin of the volunteer was chosen as the penetration object due to its lower hair level and thinner stratum corneum [53, 54]. Two-electrode measurement method was employed to record impendence during the penetration process [53–55]. An Ag/AgCl electrode was first bonded on the forearm, as shown in Fig 3. TPMA and Ag/AgCl electrodes were connected to the precision impedance analyzer (Agilent E4980A LCR Meter, Palo Alto, CA, USA). The injection voltage and frequency of the LCR meter were set at 1 V and 50 Hz, respectively. TPMA, with a force sensor (Nano 17 Titanium, ATI Industrial Automation, Detroit, MI, USA), was loaded by a linear motor (E-861, PI, Karlsruhe, Baden-Württemberg, German) towards the forearm skin at a velocity of 0.5 mm/s. The penetration force, displacement, and impendence could be simultaneously recorded by a self-developed software on the computer.

### Dry drug diffusion performance

Dry drug diffusion performance of TPMA was tested as follows: 1) TPMA microneedles were soaked in a solution of Rhodamine B (1.5 ml, 0.2 wt. %) for approximately 2 min, removed and dried for 1 h in a flow-cabinet. 2) TPMA was punctured on the New Zealand rabbit abdominal skin with approximately 5 N compression force in the vertical direction, held for approximately 3 h, and then removed. 3) The rabbit skin sample was subsequently embedded in OCT compound (SAKURA, Tissue-Tek®American), frozen at -25°C, and cut into 10-μm thick slices with a cryostat microtome (Leica, CM1850UV, Germany). 4) The skin slices were observed with an inverted fluorescence microscope (IX71, Olympus, Japan).

### In vitro permeation studies using vertical Franz diffusion cells

**Fig 4:**
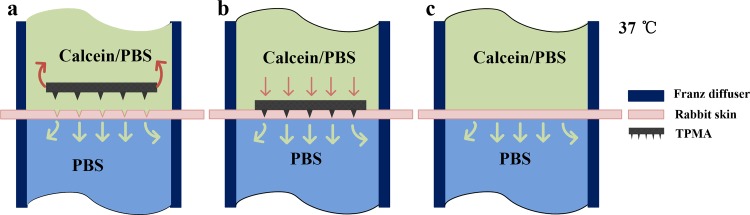
In vitro permeation studies using vertical Franz diffusion cells via (a) punctured rabbit skin, (b) TPMA with punctured rabbit skin, and (c) untreated rabbit skin.

The permeation studies of liquid calcein through rabbit skin were performed using vertical Franz diffusion cells with an orifice of 6 mm and diffusion area of 108 mm^2^ (Hellertown, PA, China). Rabbit skin samples were placed on a polystyrene foam support with stratum corneum uppermost. TPMA was pressed down manually onto the center of skin samples with an approximately 5 N compression force for 15 s by the same operator. TPMA was removed and the punctured rabbit skin was assembled in vertical Franz diffusion cells, as shown in Fig 4(A). The Franz cell consists of an upper donor chamber and lower receptor chamber with an 18-ml volume. The sampling port is an outlet connected to the receptor chamber. The donor chamber contained 4 ml of saturated calcein solution. The receptor compartment was loaded with 18 ml of PBS receptor solution. The receptor was warmed at 37°C using a built-in water circulation jacket surrounding the lower part of the Franz cells to maintain the skin surface temperature at 32°C. The receptor fluid was continuously, magnetically stirred at 280 rpm. Samples were withdrawn through the sampling port at predetermined time intervals, and the receptor chamber phase was immediately replenished with an equal volume of fresh PBS buffer to maintain a constant volume. The fluorescence intensities of samples were tested by the fluoro-spectrophotometer (FluoroMax4, HORIBA Scientific, Japan) and its concentrations were calculated as a previous paper reported [56]. At least three replicate experiments were performed. TPMA also can be pressed on the rabbit skin with a compression force of 5 N and fastened on the skin. Drug can be delivered through continuous micropores of TPMA to the dermis layer. The in vitro permeation studies using vertical Franz diffusion cells via TPMA with rabbit skin are shown in Fig 4(B) and untreated rabbit skin are shown in Fig 4(C). They all followed the same experimental procedures.

## Results and discussion

### Fabrication process and characterization

**Fig 5:**
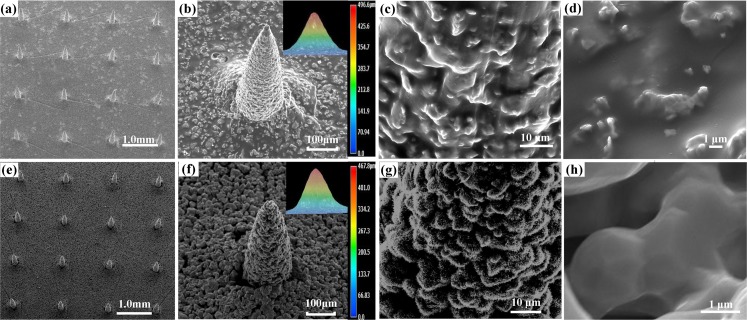
The morphology of: (a-d) TMA green body and (e-h) TPMA.

TPMA was fabricated by the modified MIM and its key steps are micro-molding of the green body and solid-state sintering of TPMA. (Fig 5A–5D) shows the green body of TMA fabricated by the micro-molding method. The micro-molding process consists of uniform filling of the PDMS mold and a drying step during which the solvent evaporates and the green body is formed. Air may remain trapped within the mold, resulting in defecs within the TMA. Therefore, the slurry in the mold was first vibrated by ultrasound and subsequently evacuated in a vacuum chamber. The slurry of TMA green body was air-dried at room temperature for approximately 48 h. The slurry drying includes diffusion of ethanol through the PDMS mold and evaporation of ethanol out of the slurry. A green body of TMA bonded by the plasticizer and binder was released from the PDMS mold, as shown in Fig 5(A). The TMA consists of 6 × 6 micro-needles with an interval of 1 mm, which matches well with that of SMA. This interval between adjacent needles can avoid the “bed of nail” effect [38]. The height of TMA is approximately 496.6 μm, which is slightly lower than the micro-needle height of SMA. It indicates that the slurry was well filled in the PDMS mold. One micro-needle of TMA is shown in Fig 5(B). The shape looks like a cone and its tip is very sharp. The diameter at the middle section is 291 μm and its tip radius is 8 μm. They are close to that of SMA. It suggests that the micro-molding method can well replicate TMA from the PDMS mold. The local surface of TMA micro-needle is presented in (Fig 5C and 5D). Its surface is relatively rough, and the plasticizer and binder fill the interstitial spaces in the titanium nanoparticle network.

The sintering process can be divided into three main stages: the debinding, sintering and cooling stages, as shown in Fig 3. During the debinding stage, the plasticizer, binder and dispersant were decomposed under Ar atmosphere of 20 mL/min. The impurity gases released from organic compounds were exhausted by Ar gas when the heating temperature increased. The compounds were removed from TMA, as shown in (Fig 5E–5H). The element distribution of TPMA would be further investigated by EDS. As the sintering temperature increased, the atoms migrated between the contact titanium nanoparticles, the titanium nanoparticles began to fuse together, the sintering neck formed and grew, and the center distance of adjacent powders decreased; meanwhile, the shrinkage and bonding strength of TPMA increased [57]. The fabricated TPMA is shown in (Fig 5E–5H). The cone shape of TPMA varies little compared with its green body. However, the micro-needles shrunk. The micro-needle tip radius, height and diameter at the middle section are approximately 20, 467.8 and 268 μm, respectively. The axial and radial shrinkages of micro-needles are approximately 6% and 8%, respectively. Therefore, the tip became blunter, height became shorter and diameter became thinner. Micropores and sintering necks can be found on the TPMA surface, as shown in Fig 5(H). The porosity of TPMA is 30.1%, and its average pore diameter is 1.3 μm. Therefore, TPMA has a lot of micropores for dry drug storage and micro-channels for liquid drug transfer or delivery. The sintering temperature and time were 1250°C and 2 h, respectively, which can guarantee the growth of sintering necks and bond strength of TPMA [58]. Furnace cooling was adopted for TPMA at a cooling rate of 2–3°C/h. The tapping temperature was about 100°C. It can somehow avoid complete oxidation of TPMA at a high temperature and ensure the hardness of TPMA.

### Impurity levels

**Fig 6:**
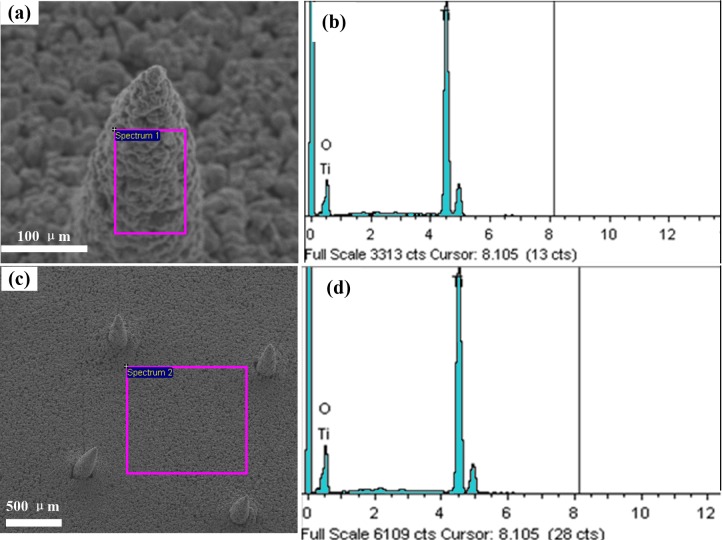
The element content of TPMA analyzed by EDS. (a-b) Elements at the surface of micro-needle and (c-d) elements on the base.

Fig 6 shows the element analysis of TPMA. Elements at the surface of the micro-needle and the base were analyzed by EDS in the area-scanning mode. There are only two elements, titanium and oxygen. The content of Ti and O is shown in Table 1. The atomic percent content of Ti and O at the surfaces of the micro-needle and base are 43/57 and 47/53, respectively. As a result, the plasticizer, binder and dispersant were decomposed during the debinding and sintering steps in the furnace. The titanium nanoparticles has a high specific surface area and pure Ti is very reactive in ambient condition, so titanium of TPMA is easily oxidized by oxygen. Titanium and its oxide have good biocompatibility and have been widely applied in medical implants [59, 60]. Therefore, TPMA may own good bio-compatibility.

**Table 1:** TPMA element content tested by EDS at the surface of the micro-needle and base.

Element	Base		Needle	
	Weight%	Atomic%	Weight%	Atomic%
**O K**	43.04	69.35	46.9	72.56
**Ti K**	56.96	30.65	53.1	27.44
**Totals**	100	100	100	100

### Penetration performance

**Fig 7:**
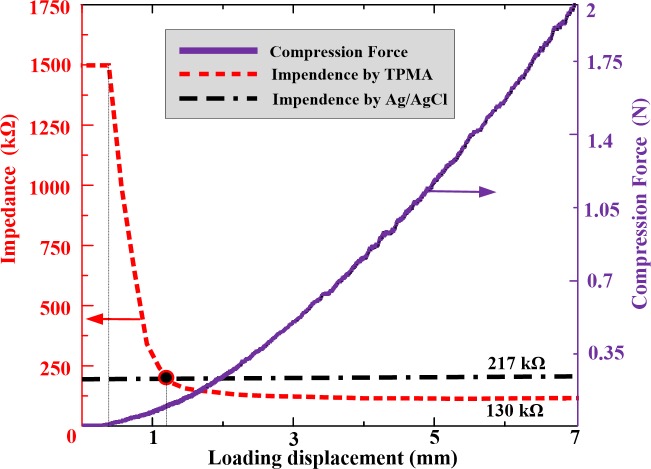
The relationship between the resistance force and impendence during the TPMA compression process.

MA can facilitate the passing of therapeutic agents through the stratum corneum by piercing the skin to create micro-channels [61]. Therefore, TPMA should have sufficient mechanical strength to penetrate the skin without breaking it. The penetration performance of TPMA is shown in Fig 7. The measured impendence can be used to judge the penetrating behavior through the skin during the compression process [55]. As TPMA moves toward the forearm skin, the impedance is extremely high and compression force is null due to the absence of contact between skin and TPMA. Once TPMA touches the skin, the compression force gradually increases with loading displacement. The impedance remains extremely high due to the high impedance of the stratum corneum layer. As the compression force is beyond 25 mN, the impendence rapidly decreases. Roxhed [62] reported the penetration force of a single microneedle in human skin was below 10 mN. It may be from the one micro-needle of TPMA penetrating through the stratum corneum and the contact impedance between TPMA and skin decreasing quickly. Subsequently, the microneedles penetrate through the skin one by one and the impendence decreases until all microneedles penetrate into skin. When the penetration force is beyond 0.4 N, the impendence reaches a steady state, its value is approximately 130 KΩ, which much lower than that measured by Ag/AgCl electrodes (217 KΩ). The average force of an adult pressing the MA with his or her thumb is approximately 20 N [63]. Therefore, we can easily press the TPMA into skin with a thumb for transdermal drug delivery. TPMA was pulled from the skin and observed with an optical microscope. None of the microneedles was ruptured during this penetration and pull process. We repeated the above test five times and micro-needles of TPMA remained intact. Therefore, TPMA has good penetration performance. However, TPMA can be bent and then broken. TPMA also has very good electrical conductivity during above impendence detection.

### Dry drug diffusion

**Fig 8:**
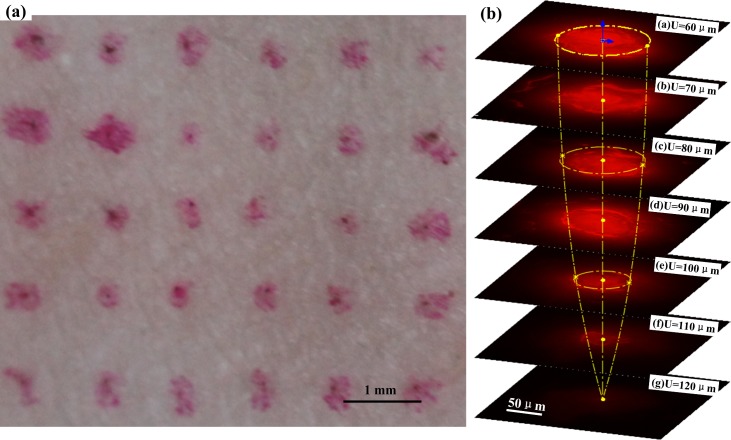
(a) The rabbit skin punctured by TPMA with Rhodamine B and (b) the drug diffusion fluorescence image of punctured skin slices at different depth.

Fig 8(A) shows the rabbit skin punctured by TPMA with Rhodamine B. The Rhodamine B dot array distributes on the surface of rabbit skin, which fits well with the arrangement of microneedles. It indicates that the microneedles were not broken during the puncture process with a compression force of 5 N. Fig 8(B) shows the drug diffusion fluorescence image of punctured skin slices at different depths. The emission fluorescence region also gradually decreases with the punctured skin depth, and its cone shape matches well with the microneedle tip of TPMA. It illustrates that the calcein stored in and on the TPMA could be well diffused to the rabbit skin, allowing TPMA to be used as a dry drug carrier. It also can demonstrate that TPMA penetrates into rabbit skin. The fluorescence intensity gradually weakens with the punctured skin depth and finally disappears at a depth of 120 μm. The layer thickness of the stratum corneum and epidermis are 10–15 μm and 50–100 μm, respectively [64, 65]; as a result, TPMA can penetrate through the stratum corneum and epidermis. TPMA will likely reach the dermis and deliver the calcein in skin. It also indirectly indicates that only about 25.6% of the microneedle length penetrates the skin, which is consistent with the statement by Martanto et al. [65, 66]. They reported that the penetration depth was 10–30% of the microneedle length. A possible explanation for the lower penetration of microneedles is that at first contact with the skin, part of the micro-needles only indents the skin, while the remaining penetrate once a sufficient compression is applied [61, 65]. The penetration depth is also related to the penetration force, micro-needle density, viscoelasticity, integrity of skin samples, and more [67].

### In vitro transdermal drug delivery

**Fig 9:**
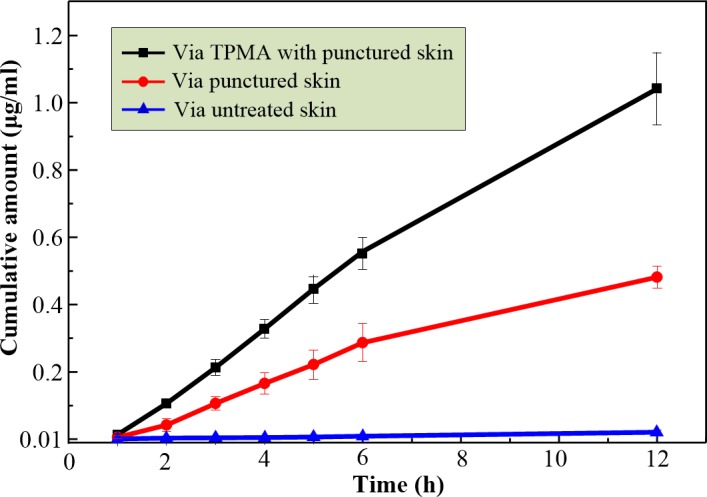
In vitro cumulative permeated amount of calcein across TPMA with punctured skin, punctured skin, and untreated skin. The results are expressed as the mean ± standard deviation of three experiments.

In vitro permeation study is a useful technique for the measurement of liquid drug transport rate through skin [65, 68, 69]. Fig 9 presents an in vitro cumulative permeated amount of calcein through skin. After 12 h of exposure, a significant difference was observed among the permeation via TPMA with punctured skin, punctured and intact skin. The cumulative permeated calcein level was 1.04 μg/mL through TPMA with punctured skin, 0.48 μg/mL through punctured skin and 0.038 μg/mL through intact skin. The corresponding flux of calcein through the punctured skin is about 12 times more than that through the intact skin. The possible explanation is that TPMA punctured through the stratum corneum and entered the dermis layer, destroying the skin barrier function. This finding has been demonstrated and discussed in Section 3.4. Several similar results were obtained in previous papers wherein solid MA was used to puncture the skin and create the micro-channels for transdermal drug delivery, enhancing the permeation level [30, 65, 67]. Thus, TPMA can be used as a puncher to create micro-channels for transdermal drug delivery. The cumulative permeated flux of calcein through TPMA with punctured skin is about twofold more than that through punctured skin and 27 times more than that through intact skin. The possible explanations are that: firstly, the open pores of TPMA are the micro-channels for drug delivery. Secondly, skin is a viscous-elastic material. The calcein can be continually transferred from a donor chamber to the dermis layer through the open pores under the gravity and capillary pressure, and collected in the receptor chamber. The micro-channels punctured by TPMA may contract and some even close as TPMA is removed [65]. It limits the permeation of drug through punctured skin. TPMA can be employed as a breaching device to deliver liquid drug through its open pores in the skin.

## Conclusions

A modified metal injection molding method was proposed to fabricate TPMA. The fabrication process, morphology, element distribution, penetration performance, dry drug diffusion and liquid drug permeation of TPMA were analyzed and discussed, and we obtained the following main conclusions:

1. The green body of TMA was replicated by micro-molding technology. The sintering process of TPMA was divided into the following three stages: debinding, sintering and cooling stages. The plasticizer, binder and dispersant were decomposed during the debinding and sintering stages under Ar atmosphere. Only Ti and O elements were left on the TPMA surface. It may guarantee the biocompatibility of TPMA. TPMA was sintered at a sintering temperature of 1250°C for 2 h. The porosity and average pore diameter of TPMA were 30.1% and 1.3 μm, respectively. The tip radius, height, diameter at the middle section of the microneedle were 20 μm, 467.8 μm and 268 μm, respectively.
2. TPMA has good penetration performance and conductivity. TPMA could easily penetrate into the rabbit skin without breaking it as the compression force was beyond 0.4 N. The impedance using TPMA and Ag/AgCl electrode at the steady state was about 130 KΩ, which was much lower than that measured by two Ag/AgCl electrodes (217 KΩ).
3. TPMA can be employed as a drug carrier to store dry drug in the pores. The dry drug can diffuse to the skin as TPMA is punctured in the skin. TPMA also may be employed as a breaching device to continually deliver the liquid drug through its open pores into the skin. The cumulative permeated flux of calcein across TPMA with punctured skin was approximately twofold more than that across punctured skin and 27 times more than that across intact skin. Therefore, TPMA may have potential for further MA-based drug delivery and could be a valuable addition to the other MA based drug delivery approaches.
